# Editorial for the Special Issue “Dietary Fibre: New Insights on Biochemistry and Health Benefits”

**DOI:** 10.3390/ijms19113556

**Published:** 2018-11-12

**Authors:** Jan Willem van der Kamp, Philip J Harris

**Affiliations:** 1 TNO Healthy Living, P.O. Box 360, 3700 AJ Zeist, The Netherlands; jan-willem.vanderkamp@tno.nl; 2 School of Biological Sciences, The University of Auckland, Private Bag 92019, Auckland Mail Centre, Auckland 1142, New Zealand

When the term dietary fibre was first coined, over sixty years ago, it only referred to plant cell walls in the diet. Since then, the definition of dietary fibre has changed considerably, and the term now encompasses a wide range of different components, including resistant starches and non-digestible oligosaccharides. As a consequence, a huge “library” of publications on dietary fibres is currently available (e.g., Stephen et al. [1]). These publications include reviews, meta-analyses, epidemiological studies, intervention studies involving humans and animals, as well as experimental studies with in vitro systems. In the current decade, most attention is being given to the key role of dietary fibres in modulating the human gut microbiome and to the study of the related effects on metabolic and mental health, which is an area of immense complexity. Contemporary research indicates that the risk of developing various non-communicable diseases is partly mediated by the structure of the gut microbiota maintained by our dietary habits. Ancestral diets, rich in plant-based foods, and dietary fibres appear to favour ecological diversity, with a richer, more complex microbiome resulting in beneficial effects for metabolic and mental health. This field is very much at its beginning stages, and many well-designed studies involving advanced analysis of the complex data generated will be required before an overall picture can emerge. This Special Issue contributes to this major goal, both with review articles and with articles describing new research. 

The review by Barbara Williams et al. [2] provides strong indications that the more complex and varied the diet is (and its ingredients), the more complex and varied the gut microbiota is likely to be, and concludes that the intake of a complex mix of plant-based foods is to be preferred over the consumption of purified dietary fibres. 

This conclusion is supported by the results of the study of Samsu U. Nurdin et al. [3]. Here, epigallocatechin-3-gallate (EGCG), the main anti-oxidant of green cincau, combined with its main dietary fibre, pectin, inhibited the ability of pectin to stimulate short-chain fatty acid production in digesta and increased cell proliferation in the distal colon. However, leaves and extracts of green cincau containing these compounds, together with many others, did not show any negative effects. 

As outlined in the review by O’Keefe (2016) [4], a dietary fibre intake of at least 50 g/day, as consumed in ancestral and non-western diets, contributes to a beneficial gut microbiome and is recommended for a substantial reduction in the risk of colorectal cancer. Suggesting effective dietary fibres as part of a diet may contribute to more detailed dietary recommendations for reducing this risk. 

The paper by Tahir Rasool Qamar et al. [5] concludes that galacto-oligosaccharides with β-1,6 and β-1,3 glycosidic linkages have dose-dependent protective effects on various biomarkers of colorectal cancer and are more protective in higher doses. This indicates that the addition of these partially synthesized, prebiotic dietary fibres can provide protective effects. 

The review by Li Pan et al. [6] indicates that a range of prebiotic dietary fibres can protect against a range of negative effects produced by the lectin soybean agglutinin, including effects on the intestinal structure, intestinal permeability, mucosal immune system, and intestinal flora. The authors indicate that functional, prebiotic oligosaccharides can bind to soybean agglutinin, thereby mitigating the negative effects of agglutinin in soybean-containing diets for monogastric animals.

The review of Abdallah Ghonimy et al. [7] addresses the important role of carnitine as a factor involved in the production of short-chain fatty acids by bacteria and the interactions between carnitine and dietary fibres, especially those with iron-binding properties, such as lignin and uronic acid-containing polysaccharides. A low dietary fibre intake can stimulate the breakdown of carnitine resulting in the production of compounds that increase the risk of atherosclerosis and cardiovascular disease. Further investigation is required to evaluate the effects of different anti-nutritional factors on carnitine bioactivity. 

In their study, Sophie Fehlbaum et al. [8] used an in vitro fermentation screening platform (i-screen) inoculated with adult fecal microbiota and exposed it to a range of different dietary fibres. They indicated that the concept of prebiotics has now been considerably broadened beyond the initial concept of non-digestible oligosaccharides and their impact on bifidobacteria and lactobacilli. Their findings support the potential for α-galactooligosaccharides, xyloologosaccharides, and oat β-glucan acting as novel prebiotics, because they caused positive shifts in microbiome composition and short-chain fatty acid production that point to potential health benefits.

Finally, another elegant in vitro study was described by Susanne Naumann et al. [9]. They investigated the interaction of dietary fibres with bile acids using simulated in vitro conditions. They compared a commonly used centrifugation method and a modified dialysis method using dietary fibre-rich materials from different sources and concluded that the dialysis method could be used to understand the influence of viscosity on bile acid release from dietary fibres. This may be due to entrapment of bile acids in the viscous chyme matrix in the in vitro conditions used. Further studies on dietary fibre structure and the mechanisms responsible for the viscous effects are required to understand the formation of entangled networks responsible for the entrapment of the bile acids.
